# Is real world evidence influencing practice? A systematic review of CPRD research in NICE guidances

**DOI:** 10.1186/s12913-016-1562-8

**Published:** 2016-07-26

**Authors:** Jessie O. Oyinlola, Jennifer Campbell, Antonis A. Kousoulis

**Affiliations:** Clinical Practice Research Datalink (CPRD), Medicines and Healthcare products Regulatory Agency, 151 Buckingham Palace Road, Victoria London, SW1W 9SZ UK

**Keywords:** Guidance, Real world evidence, Clinical practice research datalink

## Abstract

**Background:**

There is currently limited evidence regarding the extent Real World Evidence (RWE) has directly impacted the health and social care systems. The aim of this review is to identify national guidelines or guidances published in England from 2000 onwards which have referenced studies using the governmental primary care data provider the Clinical Practice Research Datalink (CPRD).

**Methods:**

The methodology recommended by Preferred Reporting Items for Systematic Reviews and Meta-Analyses (PRISMA) was followed. Four databases were searched and documents of interest were identified through a search algorithm containing keywords relevant to CPRD. A search diary was maintained with the inclusion/exclusion decisions which were performed by two independent reviewers.

**Results:**

Twenty-five guidance documents were included in the final review (following screening and assessment for eligibility), referencing 43 different CPRD/GPRD studies, all published since 2007. The documents covered 12 disease areas, with the majority (*N* =7) relevant to diseases of the Central Nervous system (CNS). The 43 studies provided evidence of disease epidemiology, incidence/prevalence, pharmacoepidemiology, pharmacovigilance and health utilisation.

**Conclusions:**

A slow uptake of RWE in clinical and therapeutic guidelines (as provided by UK governmental structures) was noticed. However, there seems to be an increasing trend in the use of healthcare system data to inform clinical practice, especially as the real world validity of clinical trials is being questioned. In order to accommodate this increasing demand and meet the paradigm shift expected, organisations need to work together to enable or improve data access, undertake translational and relevant research and establish sources of reliable evidence.

## Background

It is generally agreed that the provision of healthcare should be based on evidence, principally so that a patient receives the best advice or treatment for their condition [1]. As medical evidence is vast and at times contradictory, it is important to have a standard format which presents the evidence for a specific disease or treatment in a way that will help healthcare professionals to grasp and apply such evidence in everyday practice [2]. Standardised evidence also helps to address issues such as inappropriate variability among healthcare professionals in the provision of care [3]. Examples of this are guidelines and guidances.

Guidelines and guidances are documents which incorporate current evidence from reviewed sources in order to develop clear and comprehensive recommendations on the prevention, treatment and care of patients with specific diseases and conditions [4]. These documents can then be used by health and social care professionals to support their decision-making on the care of a patient. The benefits of using guidelines/guidances include the rapid dissemination of updates and changes in clinical practice and the ability to tailor treatment to different clinical situations [5]. Within the UK, the main body responsible for the generation and publication of guidelines and guidances is the National Institute for Health and Care Excellence (NICE) whose primary objective is to advise professionals working in the National Health Service (NHS) on how to provide the highest achievable standard of care [6].

Although the process for developing these documents has moved from being primarily expert knowledge based to being primarily evidence based, there is still concern regarding the sources of evidence. There is heavy reliance on Randomised Controlled Trials (RCTs) for generating evidence for clinical guidelines as (according to many ‘hierarchies’ of evidence) they are thought of as the ‘gold standard’ [7]. However, there are several disadvantages which make evidence from RCTs appear less practical in terms of application to patient care, a key one being the fact that RCTs are generally conducted under controlled conditions on a small number of patients over a fairly short period of time. Even if treatment proves effective in the trial, this does not mean the same effect will translate into the general population as patients in the ‘real world’ can often be more diverse in terms of age, ethnicity, gender and tend to have more comorbidities which may have an impact on the efficacy of a treatment [8]. Therefore there is a limit to the type of evidence that can be generated from RCTs to address key clinical questions which clinicians face on a daily basis [9]. Additionally, there is also the cost of running clinical trials [10] and the increased interest in obtaining return on investment in healthcare [1]. One possible solution is the use of routinely collected data or clinical databases, research outputs of which are often collectively called Real World Evidence (RWE).

Since the transition of paper healthcare records to Electronic Health records (EHRs) it has been possible to create large datasets containing important information such as clinical events, laboratory results, treatment history, etc. [11] These are often referred to as big data or Real World Data (RWD) and present several advantages to health care:

- they help to strengthen current understanding of healthcare delivery and the outcomes of patients [12],
- they greatly increase the potential of generating new knowledge as researchers can work to answer important clinical questions (which may otherwise not have been possible) [12] and
- they can support the development of evidence-based personalised medicine through the linking of EHRs to genomic datasets [13]. EHRs may also enable patients to take a more active role in their healthcare by presenting their health records to other healthcare professionals, if and when necessary [14].

Lastly (and in this case more importantly), RWD could help with the dissemination of key information by bridging the knowledge gap for clinicians and by improving the quantity and quality of evidence used in guidelines and guidances. Best evidence can only be generated when starting with the best data [4]. An example of such a database is the Clinical Practice Research Datalink (CPRD).

CPRD (previously the General Practice Research Database) is one of the largest longitudinal databases in the world containing anonymised EHR data (e.g. demographics, symptoms, behavioural factors, tests, etc.) for 11.3 million patients in the UK [15]. CPRD has been used in over 1500 observational research studies covering a variety of disease and therapeutic areas [16]. However, it is currently not known to what extent CPRD studies have been used to inform clinical practice.

In this context, this study aimed at systematically reviewing the literature to identify guidelines or guidances published from 2000 onwards in England which have referenced studies using RWD from the CPRD. The review has focused particularly on governmental organisations (in terms of data providers and guideline developers) as the UK healthcare system is one of the best integrated systems globally.

## Methods

### Operational definitions

Guidances and guidelines were categorised according to the definitions provided by the NICE.

The categories were as follows:

- NICE Clinical Knowledge Summaries: “A readily accessible summary of the current evidence base and practical guidance on best practice in respect of over 330 common and/or significant primary care presentations” [17].
- Technology appraisals guidance: “Recommendations on the use of new and existing medicines and treatments within the NHS” [18].
- Clinical guidelines: “Recommend how healthcare professionals should care for people with specific conditions” [19].

Results which did not fit these categories but focused on the delivery of medications were defined as ‘prescribing’ guidelines.

### Search strategy

This systematic review adopted the Preferred Reporting Items for Systematic Reviews and Meta-Analyses (PRISMA) guidelines [20] and was in line with the protocol agreed by all authors.

Guidelines and guidances of interest were identified by a systematic search of four databases from 1st January 2000 to 21st March 2016 (last day of search update): NICE Evidence Search, Medline PubMed, Embase and the National Clinical Guideline Centre. All four databases were searched for guideline/guidance documents referencing studies using data from CPRD using combinations of the following keywords: *“CPRD”, “Clinical Practice Research Datalink”, “GPRD”, and “General Practice Research Database*”. Indicatively for Medline the following algorithm was used: (((CPRD OR “Clinical Practice Research Datalink” OR GPRD OR “General Practice Research Database”)) AND “guideline”[Publication Type]) AND (“2000/01/01”[Date - Publication]: “3000”[Date - Publication]).

A search diary recording the search results for each database and the meeting of the inclusion/ exclusion criteria for each document was maintained. The specific inclusion criteria were as follows: 1) a UK guideline/ guidance and 2) references research using data from CPRD or GPRD. Documents were excluded if they met one or more of the following criteria: 1) Irrelevant, 2) Not written in English, 3) Not a guidance or guideline (any other primary or secondary research paper), 4) Only mentions CPRD/GPRD (e.g. as a potential source for future studies), 5) Not available (as being updated) and 6) Draft documents or in consultation. Reference lists of all studies previously identified as having met the inclusion criteria were also manually reviewed for additional relevant documents.

The search and assessment of eligibility for included studies were performed by two reviewers working independently. Any duplicate documents were consolidated. All decisions were reached by consensus, with the addition of a third reviewer where required. A relevant PRISMA flow chart was constructed to detail the number of papers retrieved and the steps undertaken.

### Data extraction

All data were extracted by two independent investigators and consensus was reached after the involvement of a third investigator where required. Full text was available for all documents. The following information was extracted for each identified document meeting the inclusion criteria: title, year of publication, disease area, the CPRD studies cited, the exact sentences referencing and the type of guideline/ guidance and the references. Each guidance document was categorised by disease area following the categories of the British National Formulary (BNF). The information was then summarised and tabulated in a standard form. In the assessment of the results, studies referenced in more than one guideline document, were treated separately as providing different evidence in each.

A purely descriptive approach was adopted for data synthesis. Sums and means were derived where appropriate. No further statistical analysis was undertaken. As this review did not include any primary research, no form of quality assessment was necessary.

## Results

### Search results

**Fig. 1:**
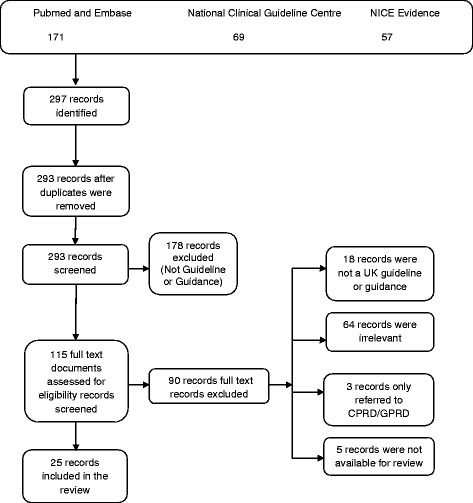
Flowchart for the systematic review following the PRISMA methodology. 297 documents in total were identified from four bibliographic databases, of which 293 were not duplicates. Following screening, 178 documents were excluded as they were not a guidance or guideline. A further 90 records were excluded based on the other exclusion criteria. 25 documents were included in the final review

The PRISMA search strategy yielded 297 documents in total from four bibliographic databases, of which 293 were not duplicates. Following screening, 178 documents were excluded as they were not a guidance or guideline. A further 90 records were excluded based on the other exclusion criteria. In total, 25 documents were included in the final review (Fig. 1), referencing a total of 43 CPRD/GPRD studies.

### Study characteristics

Baseline characteristics and detailed information of the included studies, including how the evidence from these studies was used to inform the documents, are listed in Table 1. Of the 25 documents included in the review, 12 were guidelines while the remainder (*N* =13) were guidances. The guidelines/guidances were published between 2007 and 2015 with 2008 being the only year a guidance/guideline referencing CPRD had not been published. No relevant documents were identified before 2007. The majority of documents were published in 2012 and 2015 (*N* = 7 for each year). Approximately 16 of the documents were published in the last 3 years. Of the guidelines identified, ten were clinical and the remainder were prescribing. Of the guidances identified, seven were Clinical Knowledge Summaries, two were prescribing, three were Technology Appraisals and the remainder were clinical.

**Table 1:** An overview of the studies included in the review

Guideline or Guidance and year of publication	Title	Type	Broad Disease Area	CPRD studies cited	Use of evidence from CPRD data
Guideline 2007	Guidelines for osteoporosis in inflammatory bowel disease and coeliac disease	Clinical	Gastro–intestinal system	3	Increased risk of osteoporotic fracture in patients with Inflammatory Bowel Disease (Crohn’s disease and ulcerative colitis) who use steroids; severity of disease (after adjusting for corticosteroid use) also predicted fracture [21].
					Increased fracture rates in Inflammatory Bowel Disease (Crohn’s disease and ulcerative colitis) patients with age; increased risk of osteoporotic fracture with steroid use [22].
					Small increase in fracture risk in patients with coeliac disease [23].
Guideline 2009	British Society for Rheumatology and British Health Professionals in Rheumatology guideline for the management of gout	Clinical	Musculoskeletal and joint diseases	1	Prevalence of gout [24].
Guideline 2009	Evidence-based guidelines for treating bipolar disorder: revised second edition - recommendations from the British Association for Psychopharmacology	Clinical	Central nervous system	1	Slightly greater risk of mortality from CHD and stroke in patients prescribed a higher dose of antipsychotics [25].
Guideline 2009	Guidelines (2008) for the prophylaxis and treatment of methicillin-resistant Staphylococcus aureus (MRSA) infections in the United Kingdom	Clinical	Infections	1	Incidence of community-acquired MRSA infections in the UK; association between the use of quinolone or macrolide in the year previous to community-acquired MRSA [26].
Guideline 2009	BAP updated guidelines: evidence-based guidelines for the pharmacological management of substance abuse, harmful use, addiction and comorbidity: recommendations from BAP	Clinical	Central nervous system	1	No substantive relationship between varenicline and possible adverse events (including depressed mood, agitation and suicidal thoughts) [27].
Guidance 2009	Progestogen-only Pills	Prescribing	Obstetrics, gynaecology and urinary–tract disorders	1	No effect on risk of Venous thromboembolism (VTE) with progestogens used for contraception, and not in higher doses, for the treatment of gynaecological disorders [28].
Guideline 2009	British Association of Dermatologists’ guidelines for biologic interventions for psoriasis 2009	Prescribing	Skin	1	Increased risk of herpes zoster with biologic therapy (infliximab, etanercept and anakinra) compared with DMARDs in patients with rheumatoid arthritis (RA) [29].
Guideline 2010	Chronic heart failure: Management of chronic heart failure in adults in primary and secondary care	Clinical	Cardiovascular system	1	Post-trial mortality estimates in heart failure patients [30].
Guideline 2011	Evidence-based guidelines for the pharmacological treatment of schizophrenia: recommendations from the British Association for Psychopharmacology	Clinical	Central nervous system	1	Metabolic derangements are risk factors for stroke and myocardial infarction [25].
Guidance 2012	Smoking cessation	Clinical Knowledge Summary	Central nervous system	1	No clear association between varenicline and an increased risk of fatal or non- fatal self-harm. [27]
Guideline 2012	Psoriasis	Clinical	Skin	3	Higher risk of mortality from cardiovascular disease or cerebrovascular disease in severe psoriasis patients compared to an unexposed cohort. [31]
					Higher risk of mortality from diabetes in psoriasis patients compared to an unexposed cohort; the risk of mortality from liver disease was not significantly higher [32].
					Incidence of major adverse cardiac events was higher in psoriasis patients [33].
Guidance 2012	Sore throat - acute	Clinical Knowledge Summary	Ear, nose and oropharynx	2	Low benefit of using antibiotics to prevent complications from acute sore throat [34].
					Incidence of quinsy was low but develops very quickly; low doses of antibiotics less likely to protect against quinsy [35].
Guideline 2012	Rivaroxaban for the prevention of stroke and systemic embolism in people with atrial fibrillation	Technology appraisals	Cardiovascular system	2	Prevalence of atrial fibrillation (AF) in people aged 55–64 in the UK [36].
					Event rates according to baseline level of stroke risk and the distribution of patients with different CHADS2 scores [37].
Guidance 2012	Medications in recovery: re-orientating drug dependence treatment	Prescribing	Central nervous system	1	Increased risk of mortality in the first few weeks of prescribing opioid substitution Therapy (OST); overall mortality ratio was lower in those prescribed OST than in opioid users [38].
Guidance 2012	Osteoporosis: assessing the risk of fragility fracture	Clinical	Endocrine system	2	Included in systematic review for the ‘history of falls’ as a prognostic factor for the risk of fragility of falls in Osteoporosis [39].
					Dose effect relationship between steroid use and fracture risk [40].
Guideline 2012	Infection: Prevention and control of healthcare-associated infections in primary and community care	Clinical	Infections	1	Probability of treatment failure in young women with urinary tract infections [41].
Guideline 2013	Omalizumab for treating severe persistent allergic asthma (review of technology appraisal guidance 133 and 201)	Technology appraisals	Respiratory system	1	Mortality rate for severe persistent allergic asthma patients being treated with Omalizumab [42].
Guidance 2014	Contraception - combined hormonal methods	Clinical Knowledge Summary	Obstetrics, gynaecology and urinary–tract disorders	1	Higher risk of venous thromboembolism (VTE) associated with Yasmin® than the risk of VTE with COCs containing levonorgestrel [43].
Guidance 2015	Gout	Clinical Knowledge Summary	Musculoskeletal and joint diseases	1	Prevalence of Gout [24].
Guidance 2015	Immunisations - seasonal influenza	Clinical Knowledge Summary	Immunological products and vaccines	1	No association between influenza vaccines and Guillain-Barré syndrome [44].
Guidance 2015	Otitis externa	Clinical Knowledge Summary	Ear, nose and oropharynx	1	Prescribing patterns of oral antibiotics for otitis externa [45].
Guidance 2015	Vortioxetine for treating major depressive episode	Technology appraisals	Central nervous system	1	Provided drug utilisation and healthcare resource data and comparative outcomes for diagnosed patients prescribed the specific product [46].
Guidance 2015	Multiple sclerosis	Clinical Knowledge Summary	Central nervous system	1	Prevalence of Multiple sclerosis [47].
Guideline 2015	British HIV Association guidelines on the use of vaccines in HIV-positive adults 2015	Prescribing	Infections	1	Vaccinations protect against severe disease, complications such as bronchopneumonia, hospital admission, and mortality in the elderly and those with underlying conditions [48].
Guideline 2015	Suspected Cancer: recognition and referral	Clinical	Malignant disease and immunosuppression	15	Provided the positive predictive values of symptoms for lung, oesophageal, stomach, colorectal, bladder and renal cancer to improve the diagnosis of such cancers [49].
					Provided the positive predictive values of symptoms for pancreatic cancer to improve the diagnosis of this cancer [50].
					Provided the positive predictive values of symptoms for oesophageal and stomach cancer to improve the diagnosis of these cancer [51].
					Provided the positive predictive values of symptoms for breast cancer to improve the diagnosis of this cancer [52].
					Provided the positive predictive values of symptoms for endometrial cancer to improve the diagnosis of this cancer [53].
					Provided the positive predictive values of symptoms for bladder cancer to improve the diagnosis of this cancer [54].
					Provided the positive predictive values of symptoms for renal cancer to improve the diagnosis of this cancer [55].
					Provided the positive predictive values of symptoms for myeloma to improve the diagnosis of this cancer [56].
					Provided the positive predictive values of symptoms for bladder cancer to improve the diagnosis of this cancer [57].
					Provided the positive predictive values of symptoms for urological cancer, brain cancer, CNS cancer, neuroblastoma, retinoblastoma and Wilms’ tumour in children to improve the diagnosis of these cancers [58].
					Provided the positive predictive values of symptoms for urological cancer, brain cancer, CNS cancer, leukaemia/lymphoma, Non-Hodgkin’s lymphoma, Hodgkin’s lymphoma, bone sarcoma, soft tissue sarcoma, abdominal cancer, neuroblastoma, retinoblastoma and Wilms’ tumour in children and young adults to improve the diagnosis of these cancers [59].
					Provided the positive predictive values of symptoms for brain cancer, CNS cancer, leukaemia, Non-Hodgkin’s lymphoma, Hodgkin’s lymphoma, bone sarcoma, soft tissue sarcoma neuroblastoma, retinoblastoma and Wilms’ tumour in children, young adults and adults to improve the diagnosis of these cancers [60].
					Provided the positive predictive values of symptoms for brain and CNS cancer to improve the diagnosis of these cancer [61].
					Provided the positive predictive values of symptoms for brain and CNS cancer in children and young adults to improve diagnosis of these cancers [62].
					Provided the positive predictive values of symptoms for brain, lung and CNS cancer to improve diagnosis of these cancers [63].

The guidelines and guidances covered 12 topics (grouped according to the BNF). The majority of the documents (*N* =7) were focused on diseases of the Central Nervous system (CNS). The guidances covered eight topics with the majority again focusing on diseases of the CNS. The Guidelines covered seven topics with the majority focusing on Infections, the Central nervous system and Ear, nose and oropharynx (*N* =4 for each topic). The top seven disease areas are listed in Table 2.

**Table 2:** Top seven disease areas identified in the review

Disease area	Number of guidelines and guidances
Central nervous system	7
Infections	3
Obstetrics, gynaecology and urinary–tract disorders	2
Musculoskeletal and joint diseases	2
Ear, nose and oropharynx	2
Skin	2
Cardiovascular system	2

Forty-three studies using CPRD data were referenced in the 25 documents with three studies receiving the most citations [21, 22, 24]. The guideline which referenced the most CPRD studies (*N* =15) was ‘Suspected Cancer: recognition and referral’ (2015). Only three guidances referenced more than one CPRD study (*N* =2 in all cases),’Osteoporosis: assessing the risk of fragility fracture’ (2012), ‘Rivaroxaban for the prevention of stroke and systemic embolism in people with atrial fibrillation’ (2012), and ‘Sore throat – acute’ (2012).

The evidence used from CPRD studies can be grouped into five categories. Almost three quarters provided information on disease epidemiology (*N* = 32) while the remainder were in relation to pharmacovigilance (*N* =12), pharmacoepidemiological evidence (*N* = 5), incidence or prevalence rates (*N* = 3) and health utilisation (*N* = 1).

## Discussion

### Main findings

The results of this study show RWD from CPRD have not been used to provide input for guidelines and guidances too often. The identified numbers of guidelines and guidances referencing CPRD studies seem significantly small considering that over 900 documents providing guidance have been published by NICE since its inception [64] and that CPRD has been used in over 2000 publications since it began its existence in 1987. These findings correlate to a similar study by Tricoci et al. in the USA who conducted a review of the American College of Cardiology (ACC) and the American Heart Association (AHA) guidelines in 2009. The study identified 16 relevant guidelines for their review with only 11 % of the recommendations being based on evidence from multiple sources instead of expert opinion or evidence from a single study [65]. However, the results of this review do show an increase in the frequency of RWD studies being used in guidelines/guidances in recent years. Despite the fact that clinical datasets and large longitudinal databases have been in existence for well over three decades, interest in RWD and large clinical databases for health research to influence guidelines and guidances is still in its infancy. Improvements in data processing, innovation in bioinformatics, the increased uptake of EHR systems, the increased demand for better and more efficient health care and the need for rapid generation of evidence are all contributing towards an increasing trend in its use by researchers, clinicians and policy makers [66].

### Disease areas

Identified documents covered a variety of disease areas demonstrating the breadth of research using RWD generated from EHRs. This is partly due to the range of data available through large healthcare databases and datasets and the depth of the data being enhanced by linking to other datasets.

The majority of the documents focused on diseases or treatments to do with the CNS. It appears, in this area of health, the benefits of RWE are being more utilised. For example, two of the documents refer to the treatment of patients with mental illness which correlates with research trends as several studies have investigated the patterns, drug effects and outcomes of patients used RWD [67]. The document which cited the most CPRD studies was the guideline on Suspected Cancer which highlighted the need for better methods of diagnosis and early detection and gave precise and more up-to-date information on how to detect over 200 cancers [16]. This reflects the increased interest in this disease area where several studies have looked into using EHRs to ‘flag’ recognised diagnostic clues in a timely manner [68]. This will have a substantial benefit to cancer patients as delays in diagnosis have been linked to poorer prognosis [69].

Evidence from studies using CPRD is significantly under-represented in conditions which are primarily treated in primary care (e.g. Diabetes, Obesity, Asthma, etc.). This is not because there is a lack of studies on the subject. The possible reasons for this have already been discussed above and as a result, there needs to be a review of the kind of evidence used in guidelines and guidances, for if the data is not for the purpose it was created then it is a waste of a potentially health-changing resource.

Studies using CPRD data were not referenced in guidelines in other disease areas at all, such as nutrition and blood, eye, and anaesthesia. For most of these disease areas, the reason is quite clear. CPRD currently has no or limited data on diseases and treatments that are mainly administered in secondary care (e.g. drugs administered through the eye) and there are no efficient centralised databases containing such information. However, the linking of datasets from varied health settings can provide a fuller picture of disease and health outcomes in the general population and therefore provide even more robust evidence [70]. A good example of this is the cardiovascular disease research using linked bespoke studies and electronic health records (CALIBER) dataset comprising of CPRD GOLD data and linked data from Hospital Episodes Statistics (HES), deprivation data, the Office for National Statistics (ONS) mortality information and the Myocardial Ischaemia National Audit Project (MINAP) [71]. This was a bespoke linked dataset to perform studies to improve the health of patients suffering from cardiovascular diseases and has been used in a number of useful studies, for example, to identify new associations for a range of risk factors in cardiovascular disease [72].

This shows that the ability to link datasets from a variety of sources provides immense opportunity to not only get a fuller picture of a patient’s medical history, but also investigate the interactions and associations between different treatments/diseases in different clinical settings and possibly developing predictors of health outcomes [73].

### The future

Looking towards how clinical evidence can be improved, one would directly look into the organisations providing the data. As CPRD and other data providers continue to expand their linkages to other data sources and the benefits of linked data sources increase in recognition, funds should be invested in creating datasets in all sectors of health. This will enable healthcare professionals to make sound decisions based on RWD, regardless of their line of work. The Health and Social Care Information Centre (HSCIC) who manage and maintain the balance between the sharing of information for community benefit and respecting the confidentiality and wishes of patients have been key in enabling research through linked data in the UK [74].

### Strengths and limitations

There are several strengths to this study. Firstly, it was conducted using the gold-standard method for conducting systematic reviews [75]. The PRISMA methodology has been found to improve the completeness of systematic review and meta-analysis reporting [76]. Furthermore, an exhaustive search of multiple bibliographic databases was followed. NICE and the National Clinical Guideline Centre are the main databases for UK clinical guidelines and guidances with the majority of health-related organisations referencing these sites for further information or access. The study focused on identifying research which has used data from the most widely used source of RWE (CPRD) and in a country that uses medical informatics research extensively. Lastly, the authors have the relevant experience and knowledge of CPRD, the provision of healthcare in the UK and the process for conducting systematic reviews.

However, limitations of the review need to be acknowledged. Firstly, this review focused only on guidelines and guidances which used evidence from studies using CPRD data. There are other longitudinal databases in the UK such as The Health Improvement Network (THIN) and QResearch. It also did not look into what type of data was used in each study (e.g. use of linked data). For future studies, it would be interesting to investigate whether evidence from other longitudinal databases have been used to inform guidelines/guidances. This study also focused on guidelines and guidances published for health and social care in England. Future studies could compare how RWD is used not only nationally but also internationally and identify the trends and differences that may exist. Lastly, guidelines and guidances which were currently ‘under review’ were not included in the review as they were liable to change once published. Therefore it would be worth conducting the review again at such a time when these guidelines/guidances become available to see how they affect the current results.

## Conclusions

In this systematic review, we confirmed that Real World Evidence from the Clinical Practice Research Datalink has been used inconsistently but increasingly in the last decade, to inform guidelines and guidances published in the United Kingdom. The increased uptake in recent years, noted in our results, shows that this area of healthcare is changing and this review captures a phase in this transition. To capitalise on the potential value of using Real World Evidence, researchers need to ensure they undertake research of translational value to the healthcare community. Organisations which develop guidelines should also work to identify Real World Evidence sources which will give a more realistic view of how an intervention works in actual healthcare settings. Finally, key points extrapolated from our review include increasing the quality of available Real World Evidence (which will require investment on capacity, skills and accessibility) and maintaining public trust (which will be key for wider uptake).

## Abbreviations

ACC, American college of cardiology; AHA, American heart association; BNF, British national formulary; CALIBER, cardiovascular disease research using linked bespoke studies and electronic health records; CNS, central nervous system; CPRD, Clinical Practice Research Datalink; EHR, electronic health record; HES, Hospital Episodes Statistics; HSCIC, Health and Social Care Information Centre; MINAP, myocardial ischaemia national audit project; NHS, National Health Service; NICE, National Institute for health and Care Excellence; ONS, Office for National Statistics; PRISMA, preferred reporting items for systematic reviews and meta-analyses; RCT, randomised controlled trial; RWD, real world data; RWE, real world evidence; THIN, the health improvement network
